# Impact of ROS-Dependent Lipid Metabolism on Psoriasis Pathophysiology

**DOI:** 10.3390/ijms232012137

**Published:** 2022-10-12

**Authors:** Adam Wroński, Piotr Wójcik

**Affiliations:** 1 Dermatological Specialized Center “DERMAL” NZOZ in Bialystok, 15-453 Bialystok, Poland; 2 Department of Analytical Chemistry, Medical University of Bialystok, Mickiewicza 2D, 15-222 Bialystok, Poland

**Keywords:** psoriasis, lipid mediators, ROS, eicosanoids, endocannabinoids, lymphocytes

## Abstract

Psoriasis is the most common autoimmune disease, yet its pathophysiology is not fully understood. It is now believed that psoriasis is caused by the increased activation of immune cells, especially Th1 lymphocytes. However, in psoriasis, immune cells interfere with the metabolism of keratinocytes, leading to their increased activation. Therefore, the pathophysiology of psoriasis is currently associated with the overproduction of ROS, which are involved in the activation of immune cells and keratinocytes as well as the modulation of various signaling pathways within them. Nevertheless, ROS modulate the immune system by also boosting the increasing generation of various lipid mediators, such as products of lipid peroxidation as well as endocannabinoids and prostaglandins. In psoriasis, the excessive generation of ROS and lipid mediators is observed in different immune cells, such as granulocytes, dendritic cells, and lymphocytes. All of the above may be activated by ROS and lipid mediators, which leads to inflammation. Nevertheless, ROS and lipid mediators regulate lymphocyte differentiation in favor of Th1 and may also interact directly with keratinocytes, which is also observed in psoriasis. Thus, the analysis of the influence of oxidative stress and its consequences for metabolic changes, including lipidomic ones, in psoriasis may be of diagnostic and therapeutic importance.

## 1. Psoriasis

Autoimmune diseases are a wide group of conditions that greatly affect patients’ daily life. Abnormal and chronic activation of immune cells typical to these diseases leads to chronic inflammation and damage to the body. In some cases, tissues are directly attacked by the immune system, as in e.g., rheumatoid arthritis in which lymphocytes produce antibodies against joints [1]. Nevertheless, in other cases, the pathomechanism is more complex and less understood. One such disease is psoriasis, especially its skin type called psoriasis vulgaris, in which chronic inflammation and production of different cytokines by leukocytes leads to increased proliferation of keratinocytes [2]. On the other hand, activated keratinocytes also produce cytokines, which induce leukocyte migration to the skin and activate them, leading to a positive loop between activated lymphocytes and keratinocytes. As a consequence, psoriatic plaques are formed on the skin. These plaques, which are a clinical manifestation of psoriasis, feature increased epidermal thickness due to keratinocyte hyperproliferation and increased leukocyte infiltration into the skin. A typical exanthema in psoriasis vulgaris is flat-raised papules of various sizes, clearly demarcated from the surrounding area—they are covered with a layer of scales, after scratching, which shows a shiny surface, and then punctured bleeding. Plaques most often occur on the skin of the scalp and the skin of the feet and hands [3]. Histological analysis of samples taken from skin lesions in psoriasis indicates epidermal cell hyperplasia, parakeratosis (persistent cell nuclei in the stratum corneum), and irregular thickening of the epidermis as well as infiltration of the skin by leukocytes. The severity of the disease is assessed on the PASI scale, which combines the severity (erythema, induration, and desquamation) and percentage of the affected area in the range of 0–72 (theoretically calculated maximum value).

However, in psoriasis, metabolic changes do not only affect the skin, as increased activation of peripheral blood leukocytes has also been observed in peripheral blood. In some cases, psoriasis may develop into psoriatic arthritis, in which joints are affected too [2,4].

Nevertheless, the exact pathophysiology of psoriasis is still not fully understood. It is believed that the simultaneous action of both genetic and environmental factors is necessary to develop clinical symptoms of psoriasis. These environmental factors are referred to as “trigger factors,” and the most important among them seem to include bacterial or viral infections, UV radiation, psychological stress, and smoking. The majority of these factors are associated with the overproduction of ROS, leading to oxidative stress, which may contribute to the development of psoriasis [5]. Nevertheless, injuries also may trigger psoriatic plaques. It is well known that in psoriatic patients, even a slight injury of non-affected skin leads to the formation of psoriatic plaques in the place where the injury occurs, which is called the Koebner phenomenon [6]. Still, psoriatic itch mediators, including neuropeptides, are released from the dermal nerve. This can be caused by various stimuli, which were found to be abnormally expressed in itchy psoriatic plaques, which leads to pruritus [7]. Additionally, psoriasis often leads to serious medical problems, which include not only cosmetic defects but also serious comorbidities like atherosclerosis, hypertension, ischemic heart disease, or myocardial infarction. Moreover, oxidative stress co-occurs with these diseases too [8,9,10].

Nevertheless, skin changes themselves also lead to low quality of life, psychological problems including low self-esteem, social problems, or even depression. Therefore, treating psoriasis is a real challenge for healthcare systems, especially since this disease is very common, affecting 3% of the population. In addition, despite great efforts to improve or to introduce new methods of psoriasis treatment, the therapies used today are ineffective, especially in the long run, expensive and difficult to use, and/or cause serious side effects. Therefore, only a better understanding of the pathophysiology of psoriasis can make a real contribution to the improvement of patients’ standard of living.

It is now well known that immune cells are regulated by different factors acting on the basis of different metabolic pathways. In psoriasis, dysregulation of all of the above-mentioned pathways is observed in different cells involved in its pathophysiology, such as lymphocytes, neutrophils, and dendritic cells [11,12]. ROS are extremely important during the innate immune response because the activation of neutrophils, the main cells responsible to a large extent for this response, depends on the production of ROS [13]. Nevertheless, the adaptive immune system is also regulated by ROS, and ROS and lipid mediators also modulate skin cell metabolism. Therefore, understanding the causes of oxidative stress and its role in the pathophysiology of psoriasis can make an important contribution to the development of pharmacotherapy for this disease.

## 2. ROS Impact on the Adaptive Immune System in Psoriasis

Psoriasis is known as a disease in which constant lymphocyte activation leads to increased generation of cytokines and, in consequence, to chronic inflammation. Nevertheless, it is not completely clear why lymphocytes get activated. In physiological conditions, lymphocytes are usually activated by dendritic cells, and some researchers suggest that the dysfunction of dendritic cells is the primary source of abnormal lymphocyte response. Antigen presentation is the dendritic cells’ main role. Normally, dendritic cells present microbial or other pathological antigens to leukocytes, whereby they can recognize and react to them [14]. Still, before that, dendritic cells must detect whether the antigen is pathological or whether it comes from the organism. An important role in this process is played by pattern recognition receptors, which react to common, universal factors that are present in pathogens. Examples of these receptors are TLR-7, TLR-8, and TLR-9, which respond to bacterial or viral nucleic acids, thus they can be recognized by dendritic cells [15]. Yet, it is suggested these receptors lose their selectivity in psoriasis and recognize human nucleic acids as well. In this process, some protein, produced by leukocytes, but also by keratinocytes (LL-37), may be involved [16,17]. These proteins form complexes that may activate TLRs, which leads to dendritic cell activation. As shown in animal studies, the activation of TLRs by a 5%-imiquimod leads to the development of similar symptoms as in psoriasis, which confirms the important role of dendritic cells in psoriasis development. Similarly, the human application of Aldara cream, which contains imiquimod, also leads to the induction of inflammation and the development of changes similar to psoriasis. Nevertheless, this treatment is insufficient to obtain full activation of the immune system in humans; therefore, despite the similarity of symptoms, typical psoriasis did not develop. Still, increased IL-6 and TNFα expression are observed in that case, as well as increased proliferation of keratinocytes, which suggest similarities between the imiquimod-induced changes and psoriatic plaques [18]. A population of dendritic cells are a characteristic of the skinare Langerhans Cells, and psoriasis is characterized by impaired migration of Langerhans cells which, similarly to other dendritic cells, promote the activation of lymphocytes [19]. 

### ROS Impact on the Activation and Differentiation of Th Subsets

In psoriasis, dendritic cells produce an increased amount of TNFα, which may be caused by the increased generation of ROS [20], as in vitro studies have shown that oxidative stress increases the production of IL-8 and TNFα by dendritic cells [21,22]. Additional confirmation of the importance of oxidative stress in the regulation of dendritic cells is the fact that in the presence of vitamins C and E (which are known to have antioxidant properties), dendritic cells are much less activated after the action of the pro-inflammatory factor, which was determined cytometrically on the basis of the activation of specific receptors surface, and produce smaller amounts of cytokines such as Il-1β, Il-12, TNFα, IFNγ [23]. Moreover, lymphocytes co-cultured with dendritic cells previously treated with the above-mentioned vitamins showed a lower intensity of proliferation and a shift of the response towards the Th2 characteristic, i.e., the production of IL-10, IL-4, and IL-5. It is also important that the activity of NFκB in dendritic cells after incubation with vitamins decreased [24]. Additionally, the phosphorylation of p38 MAPK kinase was reduced in the presence of vitamins, which proves that vitamins C and E inhibit the activity of the p38 MAPK pathway and apoptosis [25]. In contrast, other antioxidants, like bursopentine, inhibit NO generation and lipid peroxidation in LPS-activated dendritic cells. Additionally, in this case, the reduction of oxidative stress resulted in a decrease in the production of TNFα by dendritic cells, as well as the maturation of these cells [26]. This suggests that ROS signaling is necessary for lymphocyte activation by dendritic cells and their differentiation into the Th1 subset, which is dominant in psoriasis. 

After activation by dendritic cells, the main role of Th lymphocytes is cytokines generation (Table 1). It has therefore been observed that lymphocytes and their cytokines, especially interferon (IFNγ) and interleukin 17 (IL-17), affect other cells, leading to chronic inflammation and characteristic symptoms, such as skin plaques in psoriasis or arthrosis and limited mobility in psoriatic arthritis. In addition to the overall pro-inflammatory phenotype and activation of lymphocytes in psoriasis, different lymphocyte subpopulations play different roles, while in psoriasis, the Th1, Th17, and Th23 subsets dominate over Th2. Therefore, factors such as oxidative stress that intensify this imbalance are considered pro-psoriatic [27]. Th1 cells are the main cells responsible for the cellular response, and Th2 mediates the humoral response. This imbalance promotes cellular response [28], which is observed in psoriasis.

Th1 lymphocytes play a role in psoriasis development mainly through the production of IFN-γ, a cytokine with pleiotropic activity and multiple involvement in the pathophysiology of psoriasis. Th1-generated IFNγ stimulates the proliferation of keratinocytes. In vitro studies confirmed that supernatants obtained from T cell cultures strongly stimulate the growth of keratinocytes [29]. Moreover, as the addition of the IFN-γ inactivator inhibits this process, it is confirmed that IFNγ is what causes this effect [30]. Additionally, IFNγ stimulates antigen-presenting cells in the peripheral blood and skin to produce IL-1 and IL-23, which then activate Th17 [31]. Th17 are currently believe to be one of the most important cells involved in psoriasis pathophysiology [32]. Nevertheless, Th17 differentiation and stimulation may also be induced by IL-6 produced by fibroblasts. Th17 receptors are the main source of Il-17. Since clinical studies have shown that the IL-17 receptor inhibitor significantly reduces the symptoms of psoriasis, it is believed that this cytokine also plays an important role in the pathogenesis of psoriasis [33]. Still, lymphocytes also produce TNFα, a cytokine the higher level of which is observed in the synovial fluid of psoriasis patients and whose inhibitors alleviate the course of psoriatic arthritis [34,35].

Through IL-23 signaling, Th17 cells produce IL-17 and IL-21. Both cytokines are involved in the recruitment of neutrophils and in the formation of psoriatic lesions [32]. Moreover, IL-17 is present in serum, while Il-17 is in skin tissue in psoriatic plaques [32]. In addition to the ability to produce IL-17, stimulated Th17 cells can also produce IL-22 and IL-10 in much greater amounts than Th1 or Th2 and IFNγ [36]. It has been shown that cytokines produced by Th17 can stimulate keratinocytes to produce hBD2, S100A9, S100A7, and S100A [37,38]. IL-17, on the other hand, is divided into a number of subtypes. Clinical studies have shown that IL-17 receptor inhibitors significantly alleviate the symptoms of the disease, which is believed to confirm the role of this cytokine in the pathogenesis of psoriasis [39]. In vitro and in vivo studies have demonstrated that under the influence of IL-17, keratinocytes produce CCL-20, a chemotactic factor for lymphocytes [40]. In addition, IL-17 increases the expression of the ICAM-1 adhesion factor and the production of IL-8 and IL-6 by human fibroblasts. IL-6 stimulates the proliferation of keratinocytes, increases the production of IL-2 by T lymphocytes and activates cytotoxic T lymphocytes. Moreover, IL-6 inhibits the apoptosis of T cells, contributing to the survival of the chronic inflammatory reaction. This cytokine, together with TGFβ, is considered essential for the differentiation of Th17 lymphocytes [41]. Since IL-17, produced by Th17, stimulates the production of IL-6, it may be said that it is a positive feedback loop.

Nevertheless, Th22 cells are also elevated in people with psoriasis [40]. Differentiation of these cells is stimulated by IL-23 produced by macrophages and IL-22 produced by Th17. Additionally, Th22 cells also produce these cytokines, thus causing another positive feedback. IL-22 stimulates the proliferation of epidermal cells, the production of antimicrobial peptides in the skin [42] and the production of IL-20 in keratinocytes [43]. Among antimicrobial peptides, the production of which is promoted by IL-20, the most important are human beta-defensin 2 (hBD2) and psoriasis. These proteins increase inflammation and promote the migration of leukocytes, especially Th17 cells into the skin. Psoriasis also promotes endothelial cell proliferation [44,45]. Moreover, IL-23 produced by Th22, is of great interest due to its influence on the development of psoriasis. This cytokine is also produced by dendritic cells. It is believed to stimulate the differentiation of Th17 and Th22 lymphocytes [46]. However, it has been shown that the presence of certain mutations in the genes encoding IL-23 or IL-23R increases the risk of developing psoriasis [47]. Moreover, the injection of IL-23 into mice causes psoriasis-like skin lesions at the injection site [48].

On the other hand, Th2 lymphocytes are in opposition to those referred to above. Although their absolute number is increased, they are overbalanced by others. One of the main Th2 products is IL-4. It is responsible for the inhibition of IL-1 and IL-6 secretion; moreover, IL-4 inhibits Th17 differentiation and activation [49]. Moreover, IL-4 stimulates the action of Th2, and thus the further production of IL-4 and IL-10. IL-10 is a Th1-inhibitory cytokine that reduces the generation of pro-inflammatory IFN-γ and TNF-α [50,51].

**Table 1:** Th lymphocytes and their role in psoriasis development.

Parameters	Th1	Th2	Th17	Th22
Surface markers	CD4+ CD3+ CXCR3+ CXCR5+ IL-23R- [52]	CD4+ CD3+ CCR4+ CCR3+ CXCR3− CXCR5− [53]	CD3+ CD4+ CCR6+ CCR4+ IL-23R+ CXCR3− CXCR5− [52]	CD3+ CD4+ CCR10+ CCR6+ CCR4+ [54]
Cytokines produced	IFNγ TNFα IL-17 IL-2 [52]	IL-4 IL-5 IL-10 IL-13 [53]	IL-17 IL-17F IL-17A IL-21 IL-22 IL-26 IFN-γ [52]	IL-23 IL-22 [54]
Cytokines that cause differentiation of Th0 for this subpopulation	IL-12 [52]	IL-4 [53]	IL-23 IL-6 TGF-β [52]	IL-22 IL-23 [54]
Most important impact on psoriasis	Production of IFNγ which activates dendritic cells, Th17, Th22, and increases keratinocytes proliferation. [55]	Immunosuppressive, but their action is insufficient. [55]	Increases IL-6 and IL-8 generation by fibroblasts. [55]	Increases keratinocytes proliferation [55]

Generally, psoriatic lymphocytes feature increased activity of pro-oxidative enzymes and lower levels of antioxidants, which leads to increased ROS production and oxidative stress in these cells [12,28], which contributes to their activation. Nevertheless, the over-generation of ROS leads to the activation of compensation mechanisms such as the Nrf2 pathway [56]. Under physiological conditions, Nrf2 remains in a complex with its cytosolic inhibitor Keap1, but ROS by oxidation of the cysteine residue in Keap1 breaks down this complex, resulting in the translocation of Nrf2 to the nucleus, where it interacts with ARE (antioxidant response element) in the promoter region of many genes encoding cytoprotective genes, including antioxidant ones, which leads to their increased transcriptional activity [57]. Nevertheless, the effect of Nrf2 can also be inhibited by the nuclear inhibitor Bach1, which competes with Nrf2 for binding to ARE. Increased production of ROS was found in the lymphocytes of both psoriasis vulgaris and psoriatic arthritis. This leads to the activation of the Nrf2 pathway in both forms of psoriasis, especially psoriatic arthritis [28].

## 3. Lipid Mediators in the Pathophysiology of Psoriasis

**Figure 1:**
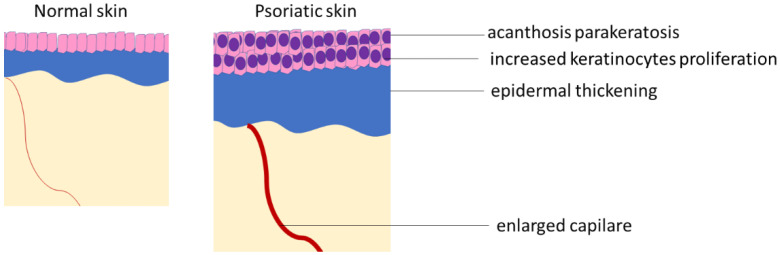
Comparison of normal and psoriatic skin.

Not only proteins undergo oxidative modifications during oxidative stress. In fact, membrane phospholipids containing polyunsaturated fatty acids (PUFA), including arachidonic, linoleic, linolenic, eicosapentaenoic, and docosahexaenoic acids [58], are the most susceptible to oxidation, which leads to the formation of lipid hydroperoxides. These are then transformed into biologically active oxidative fragmentation products, such as extremely reactive α,β-unsaturated aldehydes, including 4-hydroxynonenal (4-HNE), malondialdehyde (MDA), and other low molecular weight electrophilic aldehydes, elevated levels of which are observed in cells, psoriasis skin, but also the cells and blood plasma of psoriasis vulgaris and psoriatic arthritis patients [59,60]. 4-HNE may be also generated during enzymatic metabolism, when lipoxygenases action leads to the generation of hydroperoxyeicosatetraenoic acids (12-and 15-HpETE), which are further converted by glutathione peroxidase (GPx) to 12-or 15-hyroxyeicostetraenoic acid (12-and 15-HETE). Inactivation of GPx leads 12-and 15-HpETE to the peroxidation pathway to ultimately generate the 4-hydoxdodecadienal (4-HDDE) and 4-HNE [61]. On the other hand, phospholipids also undergo oxidative cyclization, leading to the formation of F2-isoprostanes (F2-IsoP) and neuroprostanes (Figure 1). Increased levels of free F2-IsoP and neuroprostanes are also observed in cells isolated from the blood of psoriasis patients [59]. It has been shown that 8-isoE2 and 8-isoF2 enhance the interactions between endothelial cells and macrophages and neutrophils, and thus increase the migration of these cells to sites of inflammation [62,63]. Moreover, 8-isoF2 can activate the MAPK pathways in macrophages, which leads to a higher production of IL-8 in these cells [64]. As IL-8 is important in the differentiation of Th lymphocytes into Th1, which are believed to be an important source of proinflammatory factors in psoriasis, 8-isoF2 action contributes to the development of inflammation in autoimmune diseases [65]. On the other hand, in some cases, isoprostanes can also act as anti-inflammatory agents as they react with cysteine residues of IκB kinase (IKK), thus inactivating this protein, which leads to lower activation of NF-κB [66]. On the other hand, one of the products of phospholipids oxidative fragmentation, reactive aldehyde—4-HNE—also participates in the complex regulation of inflammatory and immune responses [67]. Higher levels of generated aldehyde are also observed in the lymphocytes of patients with psoriasis vulgaris and psoriatic arthritis [12]. Moreover, significantly increased lipid peroxidation in psoriasis promotes interactions of electrophilic 4-HNE with proteins to generate adduct formation. The levels of 4-HNE-protein adducts have been shown to be approximately 2.5 times higher in the plasma of psoriatic patients compared to healthy people [68]. Still, the level of 4-HNE-protein adducts is also higher in lymphocytes, which may lead to further changes in their metabolism [12]. These changes are believed to be particularly important in the case of the transcription factor Nrf2, as the transcriptional activity of Nrf2 depends on the level and structure of its cytosolic inhibitor—Keap1, whose critical cysteine is predisposed to form 4-HNE-Cys adducts [69]. Therefore, the formation of 4-HNE-Keap1 adducts can break down the Keap1-Nrf2 complex, leading to the activation of Nrf2. As Nrf2 is believed to be an anti-oxidative and cytoprotective compound, its activation may be very beneficial in the case of psoriasis. Clinical studies, which involve fumaderm, a medicine that promotes Nrf2 activation show that its action leads to decreased PASI and better condition of patients which confirms the beneficial aspects of Nrf2 in the case of psoriasis [70,71].

**Figure 2:**
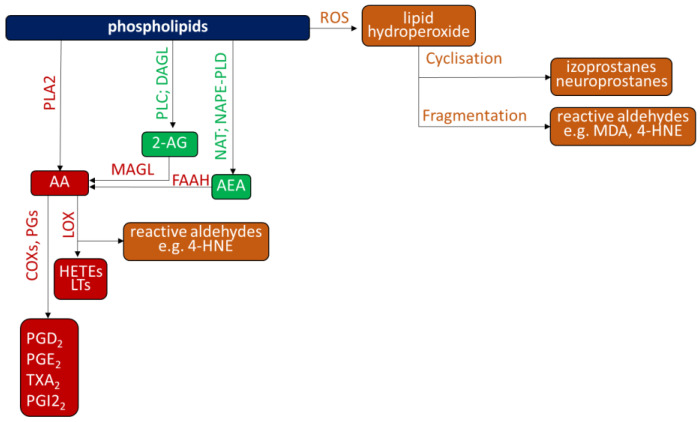
Phospholipids metabolism. 2-AG—2 acylglycerol; AEA—anandamide; COX—cyclooxygenase; DAGL—Diacylglycerol lipase; FAAH—fatty acid amide hydrolase; HETE-5—Hydroxyeicosatetraenoic acid; LOX—lipoxygenase; LTs—leukotrienes; MAGL—monoacylglycerol lipase; NAPE-PLD—N-acyl phosphatidylethanolamine-specific phospholipase D; NAT—N-acetyltransferase; PGD2—prostaglandin D2; PGE2—prostaglandnin A2; PGI2—prostaglandin I2; PGs—prostaglandin synthases; PLA2—phospholipase A2; PLC—phospholipase C; TXA2x—thromboxane A2.

Independently from lipid metabolism by ROS, lipids may undergo enzymatic metabolism, mainly by phospholipase A2 (PLA2) [72]; cyclooxygenase (COX) [73] and lipoxygenase (LOX) [74], as shown on Figure 2. In addition, it was found that PLA2 also produces bioactive metabolites with a phosphoglyceride backbone, such as lysophosphatidylcholines or platelet-activating factors [75]. On the other hand, various fatty acids undergo further metabolism with the participation of COXs and LOXs with the formation of, among others, eicosanoids, which are another large group of bioactive compounds involved in the pathophysiology of psoriasis [57]. Eicosanoids are a large group of lipid metabolites derived from PUFA peroxidation of both ω-6 (mainly arachidonic (AA) and linoleic (LA)) and ω-3 (mainly eicosapentaenoic (EPA) and docosahexaenoic (DHA)). As a consequence, patients’ phospholipid profile was studied. It showed differential expression of several classes of phospholipids [76].

### The Impact of Lipid Peroxidation Products on Adaptive Immunity in Psoriasis

It has been reported that the levels of phosphatidylcholine and phosphatidylinositols containing the above PUFAs are reduced in the lymphocytes of patients with psoriasis [12]. Moreover, it is believed that the observed significant increase in sphingomyelin content and decreased levels of phosphatidylcholine, phosphatidylinositols, phosphatidylserine, and ether-linked phosphatidylethanolamines are associated with impaired transepidermal barrier and apoptosis in keratinocytes of psoriasis patients [77]. Due to structural differences, the eicosanoid family includes two subfamilies, prostanoids (prostaglandins (PGs) and thromboxanes (TXs)) and leukotriens (LTx). Importantly, eicosanoids derived from omega-6 PUFAs including prostanoids (TXA2, PGE2, PGI2) and leukotrienes (LTB4, LTC4, LTE4) were identified as pro-inflammatory, whereas prostanoids (TXA3, PGE3, PGI3) and leukotrienes (LTB5, LTC5, LTE5) derived from omega-3 PUFAs are anti-inflammatory [78]. Due to the involvement in both pro-inflammatory and anti-inflammatory processes, eicosanoids play a key role in inflammatory signaling and may modulate skin diseases, especially psoriasis [79,80]. Both pro-inflammatory and anti-inflammatory eicosanoids were suggested as potential biomarkers of PsA, since they have been positively correlated with a joint disease score [81]. Increased levels of pro-inflammatory eicosanoids, including the oxidized lipid mediators of AA and LA, namely 8-, 12-, 15-hydroxyeicosatetraenoic acid (8-, 12-, 15-HETE) and 13-hydroxyoctadecadienoic acid (13-HODE), have been also found in skin biopsies and peripheral blood from psoriasis patients [82]. Moreover, enhanced activities of COX-1 and COX-2 in lymphocytes of patients with psoriasis vulgaris and psoriatic arthritis accompanied by a significant increase in the level of TxB2 have been also reported [83]. Importantly, TXB2 is a stable TxA2-derived metabolite that has been shown to promote the development of psoriatic dermatitis [83]. In addition to the above, the promotion of skin inflammation through NFκB-mediated metabolic pathways was previously suggested, since elevated levels of LTB4 and PGJ2 have been found in the lymphocytes of patients with psoriasis vulgaris [84].

Endocannabinoids, which are often considered elements of protective mechanisms, are the second group of lipid mediators, overproduced in skin diseases. Among them, anandamide (AEA) and 2-arachidonyloglicerol (2-AG) are the most important [85]. AEA synthesis is mediated by phospholipase D, while 2-AG is synthesized by the action of diacylglycerol lipase (DAGL) [86]. However, endocannabinoid degradation is mainly carried out by two enzymes, fatty acid amide hydrolase (FAAH) and monoacylglycerol lipase (MAGL) [86]. Nevertheless, increased activity of FAAH and MAGL was found in the keratinocytes of psoriasis patients, which led to a decrease in the levels of AEA and 2-AG [60]. Still, cannabinoids may also act as pro-inflammatory and pro-oxidative factors, depending on the receptors’ expression on target cells. As endocannabinoids are ligands for G protein-coupled membrane receptors (CB1, CB2, TRPV1, and GPR55) [86], changes in these receptors’ expression lead to changes in cannabinoids action. In most immune cells, as in the case of granulocytes, protective, antioxidative, and anti-inflammatory receptor CB2 dominates, and the pro-oxidative and pro-inflammatory CB1 expression is lower. Also in psoriasis vulgaris overexpression of CB2 receptors in granulocytes is observed [59].

**Figure 3:**
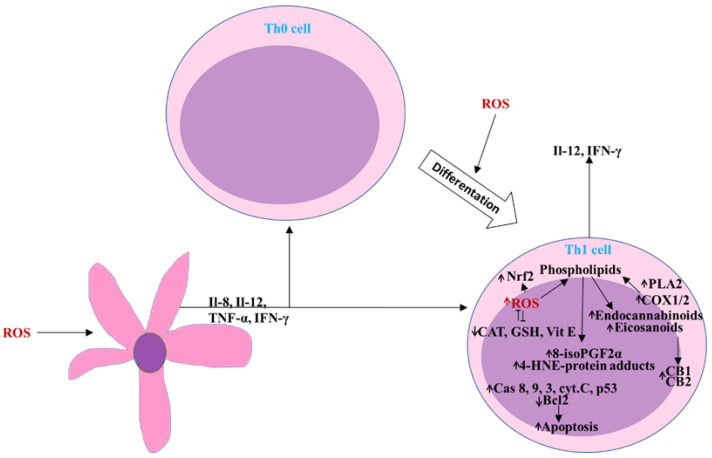
Interactions between dendritic cells and lymphocytes in psoriasis. Cas—caspase; CAT—catalase; CB1—cannabinoid receptor 1; CB2—cannabinoid receptor 2; cyt.C—cytochrom C; GSH—glutathione; IFN—interferon; IL—interleukin; ROS—reactive oxygen species; TNF—tumor necrosis factor.

Differences in the CB receptor pattern between different forms of psoriasis may be observed. In psoriasis vulgaris, an increased expression of CB2 receptors on lymphocytes was indicated, while in psoriatic arthritis, the expression of CB2 receptors was reduced despite the increase in endocannabinoid levels [12]. On the other hand, the lack of an increase in CB2 expression in psoriatic arthritis may suggest that endocannabinoid-dependent defense mechanisms are weakened. The reduction of CB2 expression with an increased CB1 receptor clearly suggests that the anti-inflammatory effect of cannabinoids is weakened, which may be a significant element in the development of common psoriasis into psoriatic arthritis. Endocannabinoids also inhibit the synthesis of pro-inflammatory cytokines (e.g., IL-6, IL-12, and IFNγ) by dendritic cells that can thus impact the differentiation of lymphocytes to Th2 and prevent their differentiation to Th1 [65], as shown on Figure 3. Moreover, it has been shown that endocannabinoids inhibit both activated Th1 and Th2 lymphocytes, whereby their addition to cells in vitro decreases their production of cytokines (TNF-α, IL-6, IL-8, IFNγ) [65]. On the other hand, it is also suggested that endocannabinoids cause a shift of the lymphocytic profile to Th2 by inducing the production of IL-4 and IL-10, which at least partially ameliorates inflammation in psoriasis [87].

## 4. ROS Impact on the Innate Immune System in Psoriasis

Since pro-inflammatory conditions dominate in psoriasis, the activation of lymphocytes activates other cells, like neutrophils, which are normally involved in the innate immune response. Therefore, activated neutrophils undergo different processes which contribute to psoriasis pathophysiology [88]. Degranulation, NETosis, and production of further cytokines seem to be the most important among them.

Neutrophils release ROS and other molecules in a process called degranulation. This process’s function is to counteract infections, but as permanent activation of neutrophils is observed in psoriasis, degranulation takes place. It has been shown that an increase in ROS levels in patients with psoriasis is accompanied by a decrease in the total antioxidant capacity in blood cells such as granulocytes, lymphocytes, and monocytes [89]. Moreover, increased ROS generation in the blood plasma and granulocytes of patients with psoriasis entails decreased levels of vitamin C, E, β-carotene, and activity of paraoxonase and superoxide dismutase as well as decreased efficiency of glutathione and thioredoxin systems and high catalase activity in plasma and circulating immune cells [59,90]. Consequently, increased ROS generation, oxidative stress, and increased lipid peroxidation are observed in psoriatic neutrophils and lymphocytes.

Still, large amounts of ROS are also produced in the NETosis process: after the neutrophil activation, large amounts of superoxide and hydrogen peroxide are converted by myeloperoxidase into hypochlorous acid and other reactive oxidants. At the same time, the neutrophil elastase migrates into the nucleus, where it degrades proteins that are necessary to maintain chromatin condensation (like H1 histones), thus causing chromatin decondensation [91]. At the same time, other histones (H2A, H3, H4) undergo citrullination by peptidylarginine deiminase 4, and the nucleus membrane disrupts. When chromatin from the nucleus is realized into the cytoplasm, the abovementioned proteins from neutrophils attach to this chromatin. Finally, the neutrophil membrane is disrupted and neutrophil extracellular traps (NETs) are formed [92]. In the case of psoriasis, an increase in the activity of pro-oxidative enzymes in neutrophils as well as an increase in the tendency to create a network of NETs by isolated neutrophils is observed. As mentioned above, NETs are also observed in skin biopsies of psoriatic patients, which confirms that this process takes place in the human body.

NETosis is highly dependent on ROS generation. Moreover, it modulates psoriasis pathogenesis in different ways. NETs, which are observed in the epidermis of skin in biopsies of psoriatic patients, stimulate keratinocytes to release inflammatory cytokines via the TLR4 and IL-36 receptor crosstalk. What is more, the chromatin of NETs, released by neutrophils, interacts with LL-37 produced by keratinocytes [93]. The IL-37 and chromatin complexes promote dendritic cells to produce pro-inflammatory mediators like IFN, IL-6, IL-12, IL-23, and TNFα, which play an important role in the initiation of the Th1, Th17, and Th22 cells’ immune response [94]. This response is further increased as proteinase 3, which is released during NETosis from neutrophils, cleaves pro-IL-36 to activated IL-36 cytokine. This cytokine, as well TNFα and IFN-γ, further activates dendritic cells. Th17 activation then leads to the production of IL-17 activating neutrophils and keratinocytes via IL-17 receptors, which generates a profound IL-17 response. The secretory leukocyte protease inhibitor (SLPI), also released during NETosis, may act in a similar way and activate dendritic cells. This is why neutrophils by NETosis play an important role, being a link between an innate and adaptive immune reaction [93].

### ROS-Dependent Protein Modifications in Psoriasis

Under the oxidative conditions associated with the development of psoriasis, proteins also undergo post-translational modifications as a result of oxidation, which causes changes in the structure and function of proteins, often resulting in significant effects on downstream cells. During the reaction of proteins with sugars/oxidized sugars, advanced glycation endproducts—AGEs are formed, which contributes to the development of diseases, including skin diseases [95]. The increased proliferation of keratinocytes observed in psoriasis results in greater demand and greater degradation of glucose by these cells [96]. Therefore, it has been suggested that AGEs also play a role in the pathogenesis of psoriasis [96]. However, skin is sensitive to changes caused by the accumulation of AGEs, which promotes increased production of ROS and consequently leads to the production of oxidized LDL in the skin [96]. Therefore AGEs may also promote chronic inflammation with activation of monocytes, macrophages, neutrophils, and endothelial cells, as these cells, when activated, produce pro-inflammatory cytokines and ROS, which may result in a molecular modification and additional production of AGEs, with further amplification of the inflammatory response [97,98]. The formation of AGEs is significantly intensified not only under oxidative stress but also in other metabolic disturbances, such as hyperglycemia and hyperlipidemia [99]. However, AGEs also activate RAGE receptors, which are found, inter alia, on the surface of keratinocytes and epithelial cells as well as DCs, monocytes, and macrophages [96]. RAGE, in turn, acts through the NF-κβ factor and tyrosine kinase [100]. In addition to AGEs, RAGE receptors also stimulate, e.g., psoriasin and HMGB1 [45,101]. Increased levels of HMGB1 have been demonstrated both in the serum of patients with psoriasis and in another autoimmune disease—rheumatoid arthritis. Additionally, in diabetes, HMGB1 levels are increased [102]. On the other hand, in vitro studies have shown that HMGB1 induces the differentiation of Th0 to Th17 cells [103]. In addition, glycated proteins can become antigens and thus enhance the immune response [104].

As in the case of lymphocytes, a higher expression of Nrf2 is also observed in the granulocytes of patients with psoriasis, both vulgaris and arthritic. Nevertheless, since the expression of HO-1, the gene considered to be the major marker of Nrf2 activation, is increased only in psoriasis vulgaris, this suggests a weakening of the effect of Nrf2 in psoriatic arthritis [59]. This may be caused by the fact that many proteins acting as Nrf2 activators, such as KAP1, p62, and p21, are elevated in psoriasis vulgaris, while their levels are lowered in psoriatic arthritis [59]. In vitro studies have shown that the level of p62 is regulated by the AP1S3 protein, and mutations in the AP1S3 gene are observed in psoriasis [105]. The increased level of p62 in psoriasis vulgaris may suggest that it promotes NFκB activation, which is confirmed by the increased level of the p52 subunit of this transcription factor, observed in psoriatic neutrophils [106]. It is known that p62 directly influences the expression of NFκB but also activates IKK, a factor responsible for the phosphorylation of NFκB and the inhibition of the NFκB inhibitor IkB [66]. As a consequence, increased expression of p62 in psoriasis vulgaris patients’ neutrophils may be responsible for the increased level and transcriptional activity of NFκB [106].

## 5. ROS Impact on Skin Cells in Psoriasis

**Figure 4:**
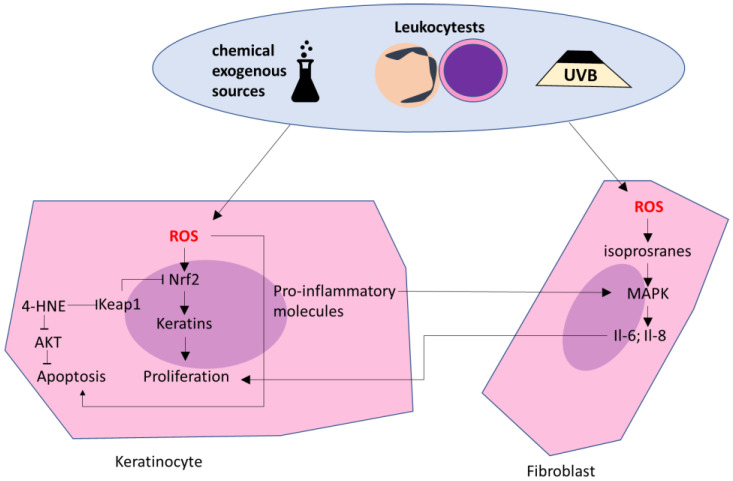
Ros impact on keratinocytes and fibroblasts in psoriasis. 4-HNE—4 hydroxynonenal; IL—interleukin; MAPK—mitogen-activated protein kinase; Nrf2—Nuclear factor-erythroid 2; ROS—reactive oxygen species; UVB—UVB radiation.

As a consequence of the abnormalities of immune cells’ functions discussed, epidermal keratinocytes are constantly stimulated to proliferation, which leads to their accumulation and, consequently, to skin changes, a characteristic symptom of psoriasis [107], which is promoted mainly by IFNγ and IL-22 and IL-23. Moreover, keratinocytes also induce the migration of leukocytes into the skin by producing chemoattractants, such as the S100 protein [37,38], which is promoted mainly by IL-17 and IFNγ. Still, as some antioxidants reduce both the production of ROS and the proliferation of keratinocytes, ROS-induced changes probably play an important role not only in immune cells but also in keratinocytes [108]. Particular attention to causing oxidative stress in psoriasis is paid to UV radiation, as the skin, which is an external organ, is more exposed than other organs to external factors, such as, among others, UV radiation. In animal studies, UV has been shown to reduce, i.e., the activity of superoxide dismutase, catalase, and glutathione peroxidase in the skin. Therefore, the use of antioxidants on the skin (in vivo) or keratinocytes (in vitro) resulted in a reduction of oxidative stress indicators, a lower level of apoptosis, and less skin damage than in the case of UV radiation [109] (Figure 4). The most important difference between UV therapy and others presented in this chapter is the fact that UV, as the only therapeutic agent, acts pro-oxidative and pro-inflammatory, but alleviates skin lesions. What is more, UV radiation is a known trigger factor that may cause the appearance of symptoms of psoriasis as well as other skin diseases. This effect is probably caused by the fact that during therapy intensity of UV is very high; therefore, the pro-apoptotic effect, dependent on ROS, becomes the dominant mechanism of UV action instead of its pro-inflammatory actions. Therefore, the intensification of apoptosis of both psoriatic and healthy keratinocytes prevent the accumulation of cells in the skin. Still, UV radiation may lead to damage in macromolecules, directly or by ROS. ROS or lipid peroxidation products and consequently modified proteins may activate the ER stress-initiated apoptotic pathway, which initiator is caspase-2, with also increased levels in UV radiated keratinocytes [110]. Therefore, UV ameliorates keratinocytes accumulation and, in this way, relieves the symptoms of the disease. Still, UV radiation may interact with both keratinocytes, and skin-infiltrating leukocytes [111,112]. Some authors suggest that UV’s impact on leukocytes, which decreases their number, is more important than its impact on keratinocytes and is responsible for its positive impact on the condition of psoriatic patients [113,114].

As mentioned above, oxidative stress leads to the activation of Nrf2, but in psoriatic keratinocytes, the activation of Nrf2 leads to a greater expression of pro-proliferative K6, K16 [13]. Additionally, in psoriatic fibroblasts, increased ROS generation and decreased antioxidant capacity are observed, which consequently results in increased levels of 8-isoprostanes and protein carbonyl groups. These changes may lead to increased MAPKs activation, and therefore increased proliferation of these cells. Additionally, this may promote the production of pro-inflammatory cytokines, such as Il-6 and Il-8 by fibroblasts, which leads to a further proliferation of keratinocytes. As increased proliferation and accumulation of keratinocytes plays an important role in the pathogenesis of psoriasis, therapies based on apoptosis regulation seem to be promising in psoriasis treatment since the increased proliferation of keratinocytes in psoriasis is not balanced by apoptosis, which leads to their accumulation [115,116]. The apoptosis process can be induced by different factors, like DNA damage, or the accumulation of abnormal proteins in the endoplasmic reticulum, leading to the activation of extrinsic, intrinsic, or endoplasmic reticulum (ER) stress-induced pathways [115,116,117]. In all of these pathways, specified caspases become activated [118]. The exact regulatory mechanisms of apoptosis are still not fully understood, but some proteins are known to activate while others inhibit apoptosis. Among them, a balance between pro-apoptotic Bcl-2-associated X protein (BAX) and B-cell lymphoma 2 (Bcl2) seems to be the most important in the regulation of the intrinsic pathway [119,120]. On the other hand, the ER stress-induced pathway is activated by damaged and non-degraded proteins [117,121]. Finally, the extrinsic pathway is activated by pro-apoptotic factors that activate death receptors, especially tumor necrosis factor receptor 1 (TNFR1) [116]. One of the three metabolic pathways leading to apoptosis, the mitochondrial pathway, is associated with increased mitochondrial membrane permeability, therefore pro-apoptotic molecules such as cytochrome C can be released from mitochondria, leading to the activation of caspases, especially caspase 9, as observed in psoriatic keratinocytes [122]. Moreover, the activation of caspases 8 and 3, associated with extrinsic and ER stress pathways, takes place in psoriasis. Nevertheless, all three of these pathways, which are activated during psoriasis, can be modulated by ROS and UV radiation. UVB activates pro-apoptotic pathways, and this effect can be associated with the fact that UVB induces a COX-2 expression, which leads to increased generation of pro-apoptotic molecules such as 15-d-PGJ2 [122].

DNA is also modified under oxidative conditions. In psoriasis, an increased level of 8-hydroxy guanosine, which is a product of oxidative modification and, at the same time, a biomarker of oxidative stress, has been demonstrated [123]. Another guanosine derivative that is formed during oxidative stress is 8-oxodG. It is known that the substitution of guanosine by 8-oxodG of the binding sequence of the transcription factor AP-1 or Sp1 reduces the binding of these factors to DNA and, consequently, disrupts their function. Nevertheless, the presence of 8-oxodG in the NFκB binding sequence does not significantly affect the binding of this factor [124,125,126,127]. Moreover, cancer is more common in psoriasis patients than in the general population [128]. It is known that oxidative stress can cause mutations in DNA, thus presumably it is the activity of ROS that may be responsible for this effect, but this has not yet been proven.

### The Role of Lipid Mediators in the Modulation of the Keratinocytes Metabolism in Psoriasis

Regardless of the effect of lipid mediators on the metabolism of blood cells of patients with psoriasis vulgaris, these mediators also impact the metabolism of basic epidermal cells—keratinocytes [82]. In the keratinocytes of patients with psoriasis, a threefold increase in the level of 4-HNE-protein adducts formed on cysteine and histidine residues of protein molecules, which are also the main amino acid connections with 4-HNE in keratinocytes of healthy people, has been demonstrated [60]. On the other hand, the adducts of proteins with 4-HNE in the keratinocytes of patients with psoriasis mainly apply to proteins with antioxidant and anti-apoptotic properties, while in the case of healthy people, the adducts created mainly concern structural proteins. These changes are believed to be particularly important in the case of the highly active transcription factor Nrf2, which is considered key in promoting the proliferation of keratinocytes in psoriasis [122]. Therefore, the formation of 4-HNE adducts with the cytosolic inhibitor Nrf2- Keap1 may further exacerbate the exfoliation of the epidermis. 4-HNE can also interact with the residues His196, His267, Cys311, and Ser473 in AKT kinase, reducing the sensitivity of AKT to phosphorylation, while modifications of Ser473, considered the main site of AKT regulation, reduce the activity of this protein [129,130]. AKT promotes cell survival, therefore, when its activity is reduced, anti-apoptotic mechanisms are activated in psoriatic cells, especially in cells exposed to the physical stressor and therapy element such as UV, which in the case of keratinocytes results in increased apoptosis, and decreased levels of p-AKT [122].

The effect of the endocannabinoid system on human keratinocyte growth and differentiation includes mechanisms of CB1 and CB2, receptor-dependent and -independent, and involves the activation of TRPV1 and PPAR receptors [131]. The activation of CB1/2, TRPVs, and PPARs in keratinocytes from psoriatic patients was observed. It has been shown that activation of the CB1 promotes the increase, while the activation of the CB2 receptor and the vanilloid receptor 1 (TRPV1) promotes the reduction of ROS and inflammatory mediators [132]. Thus, CB2 receptor agonists and CB1 receptor antagonists reduce the level of pro-inflammatory cytokines and oxidative stress [132]. In the case of PPARs, it is known that ligands of PPARβ promote skin inflammation and proliferation leading to psoriasis-like changes in mice [133]. On the other hand, ligands of PPARγ act in an anti-proliferative way and therefore may act as therapeutic factors in case of psoriasis [134].

## 6. Conclusions

ROS-induced changes may influence the pathophysiology of psoriasis in various ways. Firstly, they directly influence the metabolism of keratinocytes, leading to their increased proliferation on the one hand and to their increased apoptosis on the other. Nevertheless, since the accumulation of keratinocytes dominates in psoriasis, this suggests that the proliferation of keratinocytes has overbalanced their apoptosis. This may be due to the action of leukocytes, whereas the overproduction of ROS is observed in all major leukocytes involved in the pathogenesis of psoriasis, such as dendritic cells, lymphocytes, and neutrophils. In addition, ROS are involved in the activation of these cells and the modulation of various signaling pathways within them. Nevertheless, ROS modulate the immune system also by increasing the production of various lipid mediators, such as isoprostanes, reactive aldehydes, eicosanoids, and endocannabinoids. While isoprostanes and reactive aldehydes are generally pro-inflammatory molecules, the role of eicosanoids and endocannabinoids in modulating immunity and the pathophysiology of psoriasis is more complex. On the one hand, some eicosanoids are pro-inflammatory, but on the other, endocannabinoids are generally considered to be anti-inflammatory molecules.

Therefore, despite the impact of ROS on psoriasis being beyond doubt, their current mechanisms are still not completely known. Therefore, there is need for more research is needed in this area as it may contribute to the development of an effective therapy against psoriasis.
